# Telemedicine visits for new onychomycosis diagnoses result in fewer antifungal prescriptions than in-person care: A retrospective cohort study

**DOI:** 10.1016/j.jdin.2025.11.019

**Published:** 2025-11-29

**Authors:** Sonya V. Gupta, Kevin Chen, Christina Tang, C. William Pike, Gavin Hui, Jeremy R. Coyle, Jasmine Rana

**Affiliations:** a Stanford Dermatology, Redwood City, California; b Atropos Health, New York, New York

**Keywords:** antifungal, ciclopirox, efinaconazole, fluconazole, itraconazole, onychomycosis, tavaborole, telemedicine, terbinafine

*To the Editor:* Misdiagnosis and mismanagement of onychomycosis can have important public health and cost implications particularly in the era of increasing antifungal resistance and telehealth (TH). It remains unknown if and how onychomycosis management differs between in person (IP) and TH encounters.

Using Atropos Eos (161 M patients), we analyzed adult (>18) onychomycosis cases (2020- present) defined by first-time ICD-10 code, confirmed visit modality, and 90-day follow-up (Supplementary Fig 1, available via Mendeley at https://data.mendeley.com/datasets/5mcx5rv2rp/1). Of 98,491 eligible unique onychomycosis visits, the final matched analytic sample included 1527 TH and 1,527 IP cases (from a downsampled IP pool of 25,000), selected via 1:1 propensity score matching without replacement. High-dimensional propensity score matching with LASSO regression balanced demographics, comorbidities, and health care with LASSO hyperparameters tuned using 5-fold cross validation and 1-standard error rule.^1^^,^^2^ Primary outcomes included demographics, prescription patterns, and diagnostic testing rates by modality. Secondary outcomes included diagnostic confirmatory tests.

Baseline differences show the TH patients had lower rates of comorbidities including diabetes with complications (*P* < .001) and peripheral vascular disease (*P* = .017) compared to the IP group. After propensity score matching (Table I), the TH group was nearly twice as likely to receive no antifungal prescriptions (odds ratio [OR] 1.79, *P* < .001, E-value 1.8). Oral (OR 0.617, *P* < .001, E-value 1.6) and topical (OR 0.507, *P* < .001, E-value 2.2) antifungal prescriptions were approximately 60% and 50% less likely in the TH group, respectively, with terbinafine and ciclopirox accounting for most of the observable differences (Table II). These findings remained statistically significant 3 months post initial encounter. Although nail clippings sent for pathologic or bedside microscopy diagnosis were not able to be analyzed in this dataset, the TH group had a lower likelihood of fungal cultures being ordered (OR 0.142, *P* = .07), but this was not significant in the small number of cultures identified (number of cultures in unmatched analysis; *n* = 137, *n* = 1).

**Table I:** Demographics of propensity score matched cohorts

	In person *N* = 25000 (propensity-score matched cohort)	Telehealth *N* = 1527	*P* value∗
*N*	1527	1527	
Female (%)	891 (58)	891 (58)	>.999
Mean age (SD)	53 (15)	52 (14)	.57
Race (%)			.853
Other	614 (40)	612 (40)	
White	758 (50)	765 (50.1)	
Black	118 (7.7)	120 (7.9)	
Asian	37 (2.4)	30 (2)	
Hispanic (%)	250 (16.2)	266 (17.4)	.44
Number of encounters (SD)	2.49 (3.15)	2.24 (2.53)	.015
Comorbidity score (%)	2.26 (2.61)	2.13 (2.3)	.155
Malignancy	67 (4.4)	80 (5.2)	.272
Metastatic solid tumor	22 (1.4)	16 (1.0)	.327
Diabetes	320 (21.0)	295 (19.3)	.087
Diabetes with complications	174 (11)	123 (8.1)	<.001
Congestive heart failure	65 (4.3)	50 (3.3)	.154
Myocardial infarction	43 (2.8)	21 (1.4)	.005
Peripheral vascular disease	119 (7.8)	86 (5.6)	.017
Chronic pulmonary disease	298 (20)	308 (20)	.65
Cerebrovascular disease	67 (4.4)	68 (4.5)	.93
Dementia	12 (0.8)	8 (0.5)	.37
Hemiparaplegia	14 (0.9)	21 (1.4)	.234
Mild liver disease	95 (6.2)	110 (7.2)	.278
Severe liver disease	3 (0.2)	3 (0.2)	>.999
Renal disease	86 (5.6)	78 (5.1)	.521
Peptic ulcer disease	19 (1.2)	18 (1.2)	.869
Rheumatic disease	41 (2.7)	44 (2.9)	.741
HIV	3 (0.2)	5 (0.3)	.726

*SD*, Standard deviation.

∗ Null hypothesis rejected if *P* < .05.

**Table II:** Antifungal prescription rates by encounter type (in person or telehealth) for a first-time diagnosis of onychomycosis

	In person *N* = 1527 (%)	Telehealth *N* = 1527 (%)	OR (95% CI)	*P* value	E-value
No antifungal	1034 (67.71)	1206 (78.98)	1.79 (1.52, 2.11)	<.001	1.8
Oral antifungal	401 (26.26)	275 (18.01)	0.617 (0.519, 0.733)	<.001	1.6
Itraconazole	7 (0.46)	2 (0.13)	0.285 (0.0288, 1.5)	.179	N/A
Fluconazole	44 (2.88)	43 (2.82)	0.977 (0.638, 1.5)	.91	N/A
Terbinafine	362 (23.71)	240 (15.72)	0.6 (0.501, 0.719)	<.001	1.6
Topical antifungal	103 (6.75)	54 (3.54)	0.507 (0.362, 0.71)	<.001	2.2
Ciclopirox	74 (4.85)	30 (1.96)	0.393 (0.256, 0.605)	<.001	2.7
Efinaconazole	26 (1.7)	23 (1.51)	0.883 (0.501, 1.55)	.67	N/A
Tavaborole	3 (0.2)	2 (0.13)	0.666 (0.0556, 5.83)	1	N/A
Oral and topical antifungal	11 (0.72)	8 (0.52)	0.726 (0.291, 1.81)	.49	N/A

*CI*, Confidence interval; *OR*, odds ratio.

Reasons for lower TH oral and topical antifungal prescription rates are unclear. Possibilities include differences in disease severity, demographics, point-of-care diagnostic testing, and physical exam environments. Expert guidelines^3^ recommend testing prior to antifungal therapy, but notably more than 20% of patients in the TH group received antifungal prescriptions (Table II) with unknown diagnostic testing, raising concern for potential inappropriate therapy and highlighting an area for further investigation. Guidelines for nail TH visits do not exist, but recent data suggest they may be better suited for follow-up visits after the diagnosis is made.^4^^,^^5^

An important limitation is that clinical diagnostic codes were used with incomplete data on diagnostic testing. Disease location (eg, fingernails vs toenails), disease severity, medication interactions, and provider specialty are factors that can influence therapy and were not specified due to dataset limitations. In addition, while propensity matching controls for many observed confounders, residual confounding by unmeasured factors remains possible. Despite these limitations, our study identifies important differences in prescription patterns for TH and IP onychomycosis visits with significant cost, quality, equity, and public health implications warranting further research.

## Conflicts of interest

None disclosed.
